# 454. How Useful is SARS-CoV-2 Antigen Detection in Individuals Admitted to the Hospital With no Respiratory Symptoms?

**DOI:** 10.1093/ofid/ofad500.524

**Published:** 2023-11-27

**Authors:** Monique A Prince, Wajeeha Aiman, Modupeoluwa Owolabi, Alaa Muhanna, Evans Martin, Joe Hessell, Jihad Slim

**Affiliations:** Saint Michael's Medical Center, Newark, New Jersey; Saint Michael's Medical Center, Newark, New Jersey; Saint Michael's Medical Center, Newark, New Jersey; Saint Michael's Medical Center, Newark, New Jersey; St Georges University, Newark, New Jersey; Saint Michael's Medical Center, Newark, New Jersey; Saint Michael’s Medical Center, Newark, NJ, USA, Newark, New Jersey

## Abstract

**Background:**

Clinical findings and laboratory data have been crucial for timely diagnosis and transmission reduction of severe acute respiratory syndrome coronavirus 2 (SARS-CoV-2) which causes Coronavirus disease (COVID-19). We have observed an increase in incongruent same day antigen and reverse transcription polymerase chain reaction (RT-PCR) results in all patients presenting to the hospital. This study aims to correlate SARS-CoV-2 test results with the presence of respiratory symptoms and to find the significance of performing the antigen screening test.

**Methods:**

We conducted a single-center retrospective study, using electronic medical records of adults over 18 years of age who were tested routinely for COVID-19 with the Lyra SARS-CoV-2 RT-PCR Assay and the QuickVue SARS Antigen Test on presentation for any complaint from February 2022 to January 2023. We included all the patients who had a positive rapid antigen or RT-PCR test for COVID-19 admitted to the medical, or psychiatry unit. Patients admitted to the intensive care unit (ICU) or cardiac care unit (CCU) or those who returned a negative antigen and RT-PCR test were excluded from our study. We used the R software program to find the relative risk of pneumonia in these patients and performed pooled analysis with the random effects model.

**Results:**

Overall, 178 adults were identified with at least one test positive and were divided into three groups. In patients with positive antigen and negative RT-PCR test 20.8% had respiratory symptoms while 79.1% did not. In patients with negative antigen and positive RT-PCR, 32% had respiratory symptoms and 68% did not. In the group that returned positive for both antigen and RT-PCR tests, only 40% had respiratory symptoms and 60 % did not. Fig. 1 demonstrates the relative risk (RR) of pneumonia (PNA) in all three groups which is less than 1.

**Figure 1:**
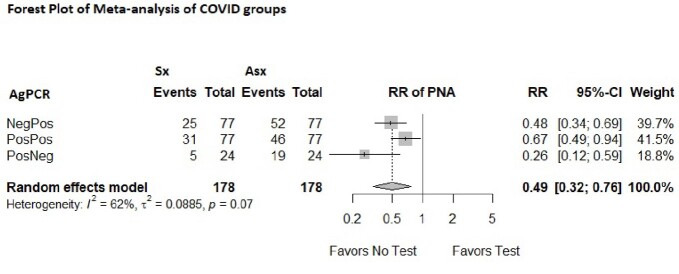
Demonstrates the relative risk of pneumonia in patients who returned a positive COVID-19 test Ag-Antigen PCR- Polymerase Chain Reaction Sx-Presence of Respiratory Symptoms Asx- Abscence of Respiratory Symptoms

**Conclusion:**

The relative risk of COVID-19 pneumonia is low in all three groups including those with a positive RT-PCR test, therefore COVID-19 antigen tests are not necessary until patients have severe respiratory symptoms or confirmed exposure. There is an ongoing need for clear guidelines for reliable detection of infection in hospitalized patients.

**Disclosures:**

**Jihad Slim, MD, FACP**, ViiV Healthcare: Advisor/Consultant|ViiV Healthcare: Grant/Research Support

